# Molecular cytogenetic analysis of the crucian carp, *Carassius carassius* (Linnaeus, 1758) (Teleostei, Cyprinidae), using chromosome staining and fluorescence *in situ* hybridisation with rDNA probes

**DOI:** 10.3897/CompCytogen.v8i3.7718

**Published:** 2014-08-18

**Authors:** Aneta Spoz, Alicja Boron, Katarzyna Porycka, Monika Karolewska, Daisuke Ito, Syuiti Abe, Lech Kirtiklis, Dorota Juchno

**Affiliations:** 1 Department of Zoology, Faculty of Biology and Biotechnology, University of Warmia and Mazury in Olsztyn, M. Oczapowskiego Str. 5, 10-718 Olsztyn, Poland; 2 Instituto Gulbenkian de Ciência, Rua da Quinta Grade, 6, 2780-156, Oeiras, Portugal; 3 Sanriku Fisheries Research Center, Department of Revitalization for Sanriku-region, Iwate University, 3-75-1, Heita, Kamaishi 026-0001, Iwate, Japan

**Keywords:** Cyprinidae, CMA_3_, FISH with rDNA, molecular cytogenetics, NOR-phenotype, polyploid species

## Abstract

The crucian carp *Carassius carassius* (Linnaeus, 1758) is a species with restricted and decreasing distribution in Europe. Six males and six females of the species from the Baltic Sea basin in Poland were examined to show sequentially CMA_3_/AgNO_3_ staining pattern, DAPI staining, and, for the first time in literature, molecular cytogenetic analysis using double-colour fluorescence *in situ* hybridisation (FISH) with 28S and 5S rDNA probes. The karyotype consisted of 20 m, 36 sm and 44 sta chromosomes, NF=156. The AgNO_3_ stained NORs were most frequently located terminally in the short arms of two sm and two sta elements, and CMA_3_-positive sites were also observed suggesting abundant GC-rich repetitive DNA in the regions. Other CMA_3_-positive sites in the short arms of six to ten sm and sta chromosomes were detected. The results based on 28S rDNA FISH confirmed the location of rDNA sites. DAPI-negative staining of NORs suggested the scarcity of AT-rich DNA in the regions. FISH with 5S rDNA probe revealed 8–14 loci (ten and 12 in respectively 49 and 29% of metaphases). They were located in two sm and eight to ten sta chromosomes and six of them were larger than others. Simultaneously, mapping of the two rDNA families on the chromosomes of *C. carassius* revealed that both 28S and 5S rDNA probes were located in different chromosomes. Molecular cytogenetic data of *C. carassius* presented here for the first time give an important insight into the structure of chromosomes of this polyploid and declining species and may be useful in its systematics.

## Introduction

The genus *Carassius* Jarocki, 1882 is a fish group of polyploid origin as are some other cyprinids of subfamilies Cyprininae and Barbinae s.l., e.g. *Cyprinus* Linnaeus, 1758 and *Barbus* Cuvier, 1816 (Vasil’ev 1985, Le Comber and Smith 2004). The importance of polyploidy in the evolution of Teleostei fishes is evident, as they are known for their advantage to survive in different environmental conditions (Gui and Zhou 2010, Yuan et al. 2010). Polyploid species are a useful model system for comparative investigations of the evolutionary process accompanied by polyploidisation at genome and chromosome level (Yuan et al. 2010, Mani et al. 2011, Pereira et al. 2012, Kumar et al. 2013, Li et al. 2014).

The crucian carp, *Carassius carassius* (Linnaeus, 1758), native to Europe, is widely distributed from the northern France to the Danube drainage and Siberia, and from England in the north to the Alps in the south. This species is adapted to both a wide range of temperature and low oxygen content and prefers densely vegetated water bodies−backwaters and oxbows of lowland rivers, and lakes (Szczerbowski and Szczerbowski 2002, Freyhof and Kottelat 2008).

The crucian carp is included in the least concern IUCN category but is regarded as disappearing in many water bodies of its range (Freyhof and Kottelat 2008). The area of distribution of this species in Poland decreased during the last two decades (Witkowski and Grabowska 2012). In recent years, interspecific hybrids have been frequently recorded between the crucian carp and the introduced Prussian carp *Carassius gibelio* (Bloch, 1782), the goldfish *Carassius auratus* (Linnaeus, 1758) and the common carp *Cyprinus carpio* Linnaeus, 1758 (Sayer et al. 2011, Wouters et al. 2012, Mezhzherin et al. 2012, Rylková et al. 2013). Hybridisation threats to the conservation of this species may lead to displacement of the genome of *Carassius carassius* by genomes of hybrids. In context of the genetic conservation of this species, it is important to determine its taxonomic diagnostic features possibly at all levels of its organisation including the chromosomal level.

The karyotype of this species has been described by Makino (1941), Chiarelli et al. (1969), Kobayasi et al. (1970), Hafez et al. (1978), Sofradžija et al. (1978), Raicu et al. (1981), Vasil'ev (1985), Vasil’ev and Vasil’eva (1985), Kasama and Kobayasi (1991) and Wang et al. (1995). For a long time there had been only two reports on the chromosomal distribution of the NORs (Mayr et al. 1986, Takai and Ojima 1986), but data involving the karyotype and some of conventional chromosome banding pattern were recently published by Knytl et al. (2013a, b).

The location of ribosomal genes in the chromosomes is commonly used as very informative cytogenetic features (Zhu et al. 2006, Zhu and Gui 2007, Singh et al. 2009; Mani et al. 2011, Pereira et al. 2012, Kumar et al. 2013). In higher eukaryotes, ribosomal RNA genes (rDNAs) are organised into the nucleolus forming major rDNA (45S) family composed of clusters of multiple copies of tandem repeated units with coding regions for 18S, 5.8S and 28S rRNA genes and non-nucleolus forming minor rDNA (5S) family (Pendas et al. 1993).

In the present study, the crucian carp *Carassius carassius* was examined for the chromosomal distribution of the nucleolar organiser regions (NORs) using sequential staining with silver nitrate (AgNO_3_), chromomycin A_3_ (CMA_3_), and DAPI staining. Moreover, fluorescence *in situ* hybridisation (FISH) with 28S (major) and 5S (minor) rDNA probes was performed. This is the first report of simultaneous localisation of two rDNA families (45S and 5S rDNA) in chromosomes of *Carassius carassius*. The ribosomal gene distribution data extend our knowledge on the cytotaxonomy and gave us information about functional structure of the chromosomes in this polyploid and declining species.

## Material and methods

### Fish specimens

In total 12 individuals, six males and six females of *Carassius carassius* (of the average length and body weight respectively, 165.0 mm and 140.0 g for females, and 151.0 mm and 124.0 g for males) were studied. They were collected from the Kortowskie Lake (53°45'43"N, 20°26'42"E), the Pregola River drainage (Baltic Sea basin) by net and then transported alive to the laboratory. Species identification followed Szczerbowski and Szczerbowski (2002) and Freyhof and Kottelat (2008). As typical for *Carassius carassius*, the specimens examined had a light (non-pigmented) peritoneum, the external morphology (deep body, rounded dorsal fin, small serration on the last unbranched ray in the dorsal fin) and general colouration (golden colour of the dorsal and lateral parts of the body).

### Chromosome preparation and staining

Mitotic chromosome preparations were made from each individual following Boron et al. (2011). First, live fish were injected with a dose of 1ml of 0.05% colchicine solution per 100g body weight. The experiments followed ethical conducts, and fish were anaesthetized using MS 222 prior to sacrificing. Mitotic chromosomes were obtained from kidney cell suspensions using the air-drying method. The kidney cells were exposed to a hypotonic solution (0.075M KCl) for 30 min and fixed in methanol: acetic acid (3:1).

Chromosomes were stained with a solution of 4% Giemsa (pH=6.8) and then classified according to Levan et al. (1964). Meta- (m) and submetacentric (sm) chromosomes were classified as biarmed, whereas subtelo- and acrocentric (sta) as uniarmed elements. Chromosomes were counted in at least 20 metaphase figs in each individual and were analysed using MultiScan software with the additional Karyotype supplement.

Chromosome slides of three males and three females were sequentially stained with AgNO_3_ and CMA_3_ according to Sola et al. (1992). The active AgNOR sites and CMA_3_-positive sites were counted in 15 metaphase figs from each individual, using MultiScan software with the additional Karyotype supplement.

### Probes and fluorescence *in situ* hybridisation (FISH)

Single colour FISH with human 28S rDNA probe or double-colour FISH with loach 5S and human 28S rDNA probes were used according to Fujiwara et al. (1998) and Boron et al. (2009). The 5S rDNA probe was labelled with biotin-16-dUTP using Biotin-Nick Translation Mix kit (Roche), while the 28S rDNA probes were labelled with digoxigenin-11-dUTP using the DIG-Nick Translation Mix kit (Roche), according to the manufacturer’s instructions. The chromosome slides were initially incubated with RNase for 60 min at 37 °C in a moist chamber. After denaturation for 1 min in 70% formamide (FA)/2×SSC, chromosome slides were dehydrated in an ethanol series, 70% for 5 min and 80%, 90%, and 100% for 2 min, each at 20 °C. Hybridisation with a mixture containing denatured rDNA probes, Bovine Serum Albumin, 50% dextran sulphate, 20×SSC, and double-deionised water was performed at 37 °C in a moist chamber. Post-hybridisation washes were performed in 50%FA/2×SSC at 37 °C for 20 min, 2×SSC and 1×SSC for 20 min each, and 4×SSC for 5 min. 5S and 28S rDNA probes were detected with Avidin-Fluorescein (Roche) and Anti- Digoxigenin-Rhodamine (Roche), respectively. Then, chromosomes were counterstained with DAPI in Antifade solution (Vector Laboratories). We show here both single colour and dual colour FISH with rDNAs because firstly prepared single colour FISH revealed DAPI banding pattern. This pattern turned out to invisible after dual colour FISH.

Hybridisation signals in at least 15 metaphase figs of each individual were observed under a Nikon Eclipse E800 fluorescence microscope using a Nikon B-2A filter for a single colour FISH and black and white CCD camera Pixera Penguin 150CL-CU (Pixera), and a Nikon Eclipse 90i fluorescence microscope equipped with ProgRes MFcool camera (Jenoptic) for capturing the images of a dual colour FISH. The images were processed using Penguin Mate ver. 1.0.8. software for RGB pseudocolour imaging (Pixera) and Lucia ver. 2.0 (Laboratory Imaging).

Voucher specimens were preserved frozen and deposited at the Department of Zoology, University of Warmia and Mazury in Olsztyn, Poland.

## Results

### Karyotype and banding patterns

**Figure 1.**
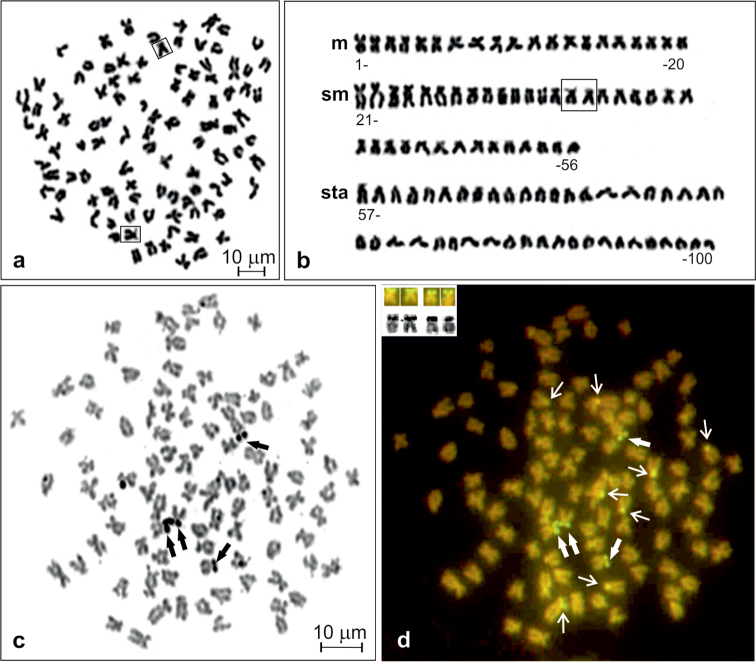
Giemsa stained metaphase (**a**), corresponding karyotype of *Carassius carassius* (**b**), and metaphase spread sequentially stained with AgNO_3_ (**c**) and CMA3 (**d**). NOR chromosomes shown in frames (in **a** and **b**), Ag-NORs and corresponding CMA3-positive sites shown by thick arrows (in **c** and **d**) and shown in inset (in **d**), other CMA3–positive sites shown by thin arrows (in **d**).

The crucian carp from the Kortowskie Lake exhibits a diploid chromosome number of 100 (Fig. 1a) without any supernumerary chromosomes in 369 (94.4%) out of 391 analysed metaphase figs. The karyotype consisted of 20 m, 36 sm and 44 sta chromosomes (Fig. 1b). The chromosome arm number (NF) was counted as 156. The first submetacentric pair (11th pair) was easily recognisable in all metaphase figs, being the largest elements in the chromosome complement. No variability in the chromosome formula was observed and heteromorphic sex chromosomes were not detected.

AgNO_3_ stained active nucleolus organiser regions (AgNORs) were located terminally at the short arms of two sm and two st chromosomes (Fig. 1c). After sequential staining with CMA_3_, all signals were observed as a distinct bright fluorescence, suggesting abundant GC-rich repetitive DNA sequences in the regions (Fig. 1d). Among 90 metaphases of six individuals, two to four CMA_3_-positive sites corresponding to AgNORs were detected, but 57.8% of metaphases showed four bright signals. Three and two such sites were observed, respectively, in 31.1 and 11.1% of analysed metaphases. In addition, there were extra CMA_3_-positive sites located at the short arms of six to ten sm and sta chromosomes. Most frequently (in 52.2% of metaphases) eight such sites at four of each of sm and sta elements (Fig. 1d) or six (in 32.2% of metaphases) sites at three of each of sm and sta were observed.

One of the submetacentric chromosomes (chromosome no. 35 of pair 18, shown in frame in Fig. 1a–b), possessing clearly visible secondary structure along its short arm, was easily distinguishable among others in all metaphase figs stained with Giemsa.

**Figure 2.**
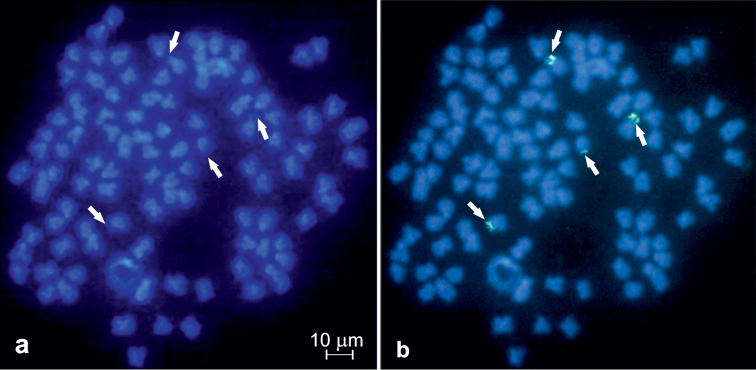
Metaphase fig of *Carassius carassius* DAPI stained (**a**) and with a single colour FISH (**b**) with 28S rDNA probe. 28S rDNA hybridisation signals shown by arrows.

**Figure 3.**
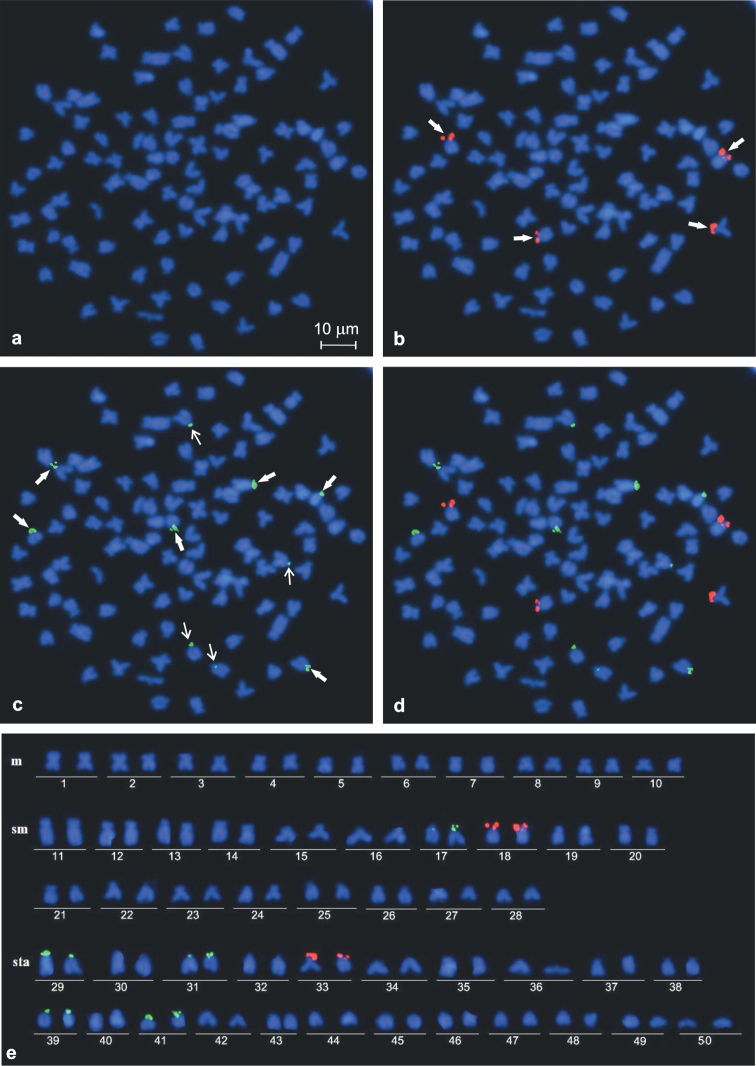
Representative mitotic metaphase figs (**a−d**) and corresponding karyotype of *Carassius carassius* (**e**): **a** DAPI stained and **b−d** most frequent hybridisation pattern after dual colour FISH with four 28S rDNA sites (**b**), ten 5S rDNA sites (**c**) and both rDNA probes (**d**). Six stronger and four weaker 5S rDNA hybridisation sites (**c**) shown by thick and thin arrows, respectively.

DAPI-counterstained chromosomes have shown some slightly visible AT-rich pericentromeric heterochromatic regions of 12–14 sta and at the short arms of four to six sm (Fig. 2a–b). However, they were not detected in the metaphase figs after using dual colour FISH that such the chromosomal regions were dimly DAPI-stained (Fig. 3a).

### FISH mapping of 28S rDNA loci

Single FISH using 28S rDNA probe analysed in 68 metaphase figs of two females and two males and dual colour FISH analysed in 243 metaphase figs of four females and four males revealed either three or four loci in their chromosome complement. In most of the metaphase figs (76.2%), the signals were found in the short arms of two each of sm and st chromosomes (Figs 2b, 3b). Three hybridisation sites were observed commonly as intense and large signals, whereas the signal in the fourth site was smaller and weaker than the other three sites. DAPI-negative staining of the observed NORs suggested the scarcity of AT-rich DNA in the regions (Figs 2a, b, 3a, b). In the rest of the analysed metaphase figs (23.8%), three 28S rDNA sites were observed in the short arms of two sm and one sta elements. Numerous metaphases showed close association of NORs involving two or sometimes three chromosomes.

### FISH mapping of 5S rDNA loci

FISH with 5S rDNA probe analysed in 243 metaphase figs of four males and four females revealed an unexpectedly large number of loci, from eight to 14. The obtained hybridisation signals had different intensities on various chromosomes and could be classified as strong and weak (Fig. 3c–d). All individuals frequently showed 10 (Fig. 1c) or 12 such loci in respectively 48.6% and 29.2% of metaphase figs. They were located at the short arms of two sms (pair 17 in Fig. 3e) and at the short arms or in a subcentromeric position of eight to ten sta chromosomes (pairs 29, 31, 39 and 41 in Fig. 3e). Six hybridisation sites of 5S rDNA were stronger than the other four to six (Fig. 3c–e). Among 15.2% and 7.0% of the rest of metaphase figs, the 5S rDNA loci were located, respectively, in eight and 14 chromosomes. Usually, in metaphase figs containing 14 signals, two signals were very weak.

Thus, *Carassius carassius* was characterised by the modal number of ten 5S rDNA loci. Signal heteromorphism was detected on the homologous chromosome of pairs 17 and 31 (Fig. 3e). Both classes of rDNA probes were always located in different chromosomes and co-localisation in the same chromosome was not observed (Fig. 3d).

No sex-dependent variability in the cytogenetic features was found.

## Discussion

Undoubtedly, the crucian carp *Carassius carassius* possesses 2n=100 chromosomes in its somatic cells but data on the karyotype reported in literature somewhat differ (Table 1). The reason for this could be that the karyotype of the crucian carp contains a lot of very small chromosomes which are similar in size. The problem mainly concerns discrimination between sm and sta chromosomes as it occurs in the karyotype of a related species *Carassius gibelio* (Boroń et al. 2011). The karyotype obtained in the present study with a larger number (56) of biarmed than (44) of uniarmed chromosomes is the same as that supposed by Knytl et al. (2013a, b). Similar karyotype characterised by the largest sm pair was described by Kobayasi et al. (1970), Sofradžija et al. (1978), Hafez et al. (1978), and Kasama and Kobayasi (1991) in *Carassius carassius*.

**Table 1.** Cytogenetical data of the crucian carp, *Carassius carassius*. Symbols of chromosomes: m – metacentric, sm – submetacentric, sta – subtelo- to acrocentric, NF – number of chromosome arms.

L.p	Locality	2n	Karyotype	NF	Cytogenetic features	Reference
1.	-	94	-	-	-	Makino 1941
2.	-	104	20m+72sm+12a	196	-	Chiarelli et al. 1969
3.	the Netherlands (Baltic basin)	100	20m+40sm+40a	160	-	Kobayasi et al. 1970
4.	France (Garonne drainage)	100	20m+44sm+36a	164	-	Hafez et al. 1978
5.	Drina R. (Danube), Bosnia	100	52m, sm+48sta	152	-	Sofradžija et al. 1978
6.	Danube R., Romania	50	20m+12sm+18sta	82	-	Raicu et al. 1981
7.	Water bodies in Moscow region, Russia (Volga drainage)	100	48m, sm+52sta	148	-	Vasil'ev 1985, Vasil’ev and Vasil’eva 1985
8.	Elbe R., Czech Republic	100	-	-	AgNOR	Mayr et al. 1986
9.	the Netherlands (Baltic basin)	100	20m+40sm +40a	160	-	Kasama and Kobayasi 1991
10.	Tarim R., Xinjiang, China	100	32m+34sm+34sta	166	-	Wang et al. 1995
11.	Elbe R., Czech Republic	100	20m+36sm+44sta	156	C bands, AgNOR, DAPI/CMA_3_	Knytl et al. 2013a, b
12.	Kortowskie Lake, Pregola R. drainage, Poland	100	20m+36sm+44sta	156	AgNOR/CMA_3,_ 45S and 5S rDNA (FISH)	present study

The crucian carp and other *Carassius* species distributed in Europe were recognised as monophyletic lineages (Rylková et al. 2013). Among them, only *Carassius carassius* is characterised by 2n=100 chromosomes and can be easily identified morphologically (Szczerbowski and Szczerbowski 2002). Identification of other species according to the chromosome number is complicated by the occurrence of both diploid and triploid specimens within *Carassius gibelio*, *Carassius langsdorfii* Temminck & Schlegel, 1846 and *Carassius auratus* (Linnaeus, 1758) (Rylková et al. 2013).

Most of the cyprinid species, for example those from the subfamilies Leuciscinae, Gobioninae and Danioninae, are characterised by 2n=50 or 2n=48 chromosomes (e.g. Vasil’ev 1985, Rab and Collares-Pereira 1995). The polyploids exist in the subfamily Cyprininae s.l., within the following tribes recognised by Yang et al. (2010): cyprinins (e.g. *Cyprinus* and *Carassius*), barbins (e.g. *Barbus* and *Tor* Gray, 1834) and oreinins (e.g. *Schizothorax* Heckel, 1838). The karyotype of *Carassius carassius* is similar to other polyploid cyprinin species (Le Comber and Smith 2004, Singh et al. 2009, Mani et al. 2011) possessing 100 chromosomes may be shown as 2n=4X=100. We assume, following Vasil'ev (1985) and Buth et al. (1991), that the haploid number of chromosomes equals n=25, so, they are tetraploids. It would be expected that the chromosomes in these species formed tetravalents during the prophase of meiosis I. Occurrence of only bivalents indicates the 'diploid nature' of *Carassius carassius*. So, this species as some others of the genera mentioned above have evolved via formation of polyploids and subsequent diploidisation process (Vasil'ev 1985, Buth et al. 1991).

The number of four AgNORs (two sm and two st) characterises the karyotype of *Carassius carassius* (Knytl et al. 2013b), but they varied from two to four as was shown in this study consistently with their transcriptional activity during the preceding interphase. Intraspecific and intraindividual variation of AgNORs results from that Ag-staining solely detects the products of active 18S, 5.8S and 28S rDNA expression in the preceding interphase (Reeder 1990).

We documented that the AgNOR sites were CMA_3_ positive similar to what is found in many other Teleostei (Knytl et al. 2013b). It can be interpreted as a high copy number of repeating units of rDNA (Gromicho and Collares-Pereira 2007). The obtained results support the hypothesis that CMA_3_ staining of GC-rich heterochromatin shows all active and non-active NORs in the chromosomes. However, the only four of numerous GC-rich DNA heterochromatin sites in the karyotype of *Carassius carassius* were associated with major ribosomal sites. The CMA_3_-positive sites being NOR-negative may be related to nucleolar dominance phenomenon reported in other organisms and other taxa of Teleostei, and in some hybrids and species of hybrid origin (Gromicho et al. 2005). The additional CMA_3_-positive sites were not found in *Carassius carassius* by Knytl et al. (2013b).

The karyotype of *Carassius carassius* after DAPI staining described by Knytl et al. (2013b) was uniform. We gained slightly visible AT-rich heterochromatic regions of DAPI-counterstained chromosomes in single colour FISH staining, whereas the chromosome DAPI differentiation was not revealed using dual colour FISH. The differences may result from the level of chromatin condensation and/or technical reasons. DAPI-negative staining of the NORs reported here and described in, for example, *Rhodeus amarus* (Bloch, 1782) (Kirtiklis et al. 2014) reflected the occurrence of GC-rich heterochromatin and the scarcity of AT-rich DNA in these regions.

The results from FISH with 28S rDNA confirmed for the first time in literature that the karyotype of *Carassius carassius* (2n=100) is characterised by the conservative number of NORs − four − located in the short arms of two sm and two st chromosomes. It was mentioned by Knytl et al. (2013b) that this NOR chromosomal pattern supported a hypothesis of the palaeotetraploidy of the crucian carp genome as was earlier suggested by Vasil’ev and Vasil’eva (1985). Similarly, five located NORs were found in the karyotype of a related species *Carassius gibelio* with 162 chromosomes (Zhu and Gui 2007). According to Foster and Bridger (2005), the terminal position of 45S rDNA, considered as a primitive stage in Teleostei, would promote chromosomal dispersion due to their proximity within an interphase nucleus. The presence of a single chromosomal pair bearing 28S rDNA was assumed to represent an ancestral condition in fishes, since this pattern had been reported in species representing all so far investigated fish orders (Martins and Wasko 2004, Nakajima et al. 2012). Taking this into consideration the presence of two pairs of NORs found in *Carassius carassius* may be connected with the polyploid origin of the species. A similar pattern with two or more pairs of NOR chromosomes is known in species from the genus *Tor* (2n=100) (Singh et al. 2009, Mani et al. 2011). However, two or multiple NORs were observed in many other non-polyploid cyprinid species with 2n=50 chromosomes (Pereira et al. 2012, Kumar et al. 2013).

The weak or missing signal of hybridisation in one out of the four NORs in the karyotype of *Carassius carassius* could be due to either a low copy number of 28S rDNA or a deletion of these genes, or due to technical reasons (Pendas et al. 1993, Fujiwara et al. 1998). Alternatively, it could be an effect of chromosomal rearrangement associated with the occurrence of transposable elements (Pearson et al. 2005). Rapid chromosome rearrangement was proposed as exiting in the postpolyploidy genome of *Carassius gibelio* according to size variation and 45S rDNA distribution (Zhu and Gui 2007).

The FISH localisation of the 5S rDNA revealed that these sequences are spread in at least eight chromosomes. *Carassius auratus* (2n=100) is characterised by 5S rDNA large hybridisation sites located at the short arms of two st and from two to eight smaller 5S rDNA sites whereas a triploid form of *Carassius gibelio* (3n=162) had three larger sites and from six to 18 small ones (Zhu et al. 2006). Strong signals of 5S rDNA at the short arms of two to four pairs of acrocentric or subtelocentric and several additional weak signals were also observed in the karyotype of *Cyprinus carpio* (Inafuku et al. 2001). Multiple loci for the 5S ribosomal sequences and their varying hybridisation signals seem to be typical for *Carassius* and *Cyprinus* species. However, the location of 5S rDNA sites in the karyotypes of *Carassius carassius*, *Carassius auratus* and *Carassius gibelio* does not confirm the opinion about conservative pattern of 5S rDNA loci distribution in closely related species (Gromicho et al. 2006, Singh et al. 2009, Mani et al. 2011).

Commonly in Teleostei, there is a single locus for the 5S ribosomal sequences, which is regarded as an ancestral condition while the hybridisation pattern with two or more loci may be considered as a derived state (Martins and Wasko 2004, Singh et al. 2009, Nakajima et al. 2012, Kumar et al. 2013). Apart from the above mentioned species, two and more loci of 5S rDNA were found also in some natural hybrids and/or polyploid taxa (Martins and Wasko 2004, Gromicho et al. 2006, Mani et al. 2011, Pereira et al. 2012) as well as in some diploid species (Kirtiklis et al. 2010). This requires verification whether the numerous chromosomes containing the sequence of 5S rDNA are an idenfining marker of species which are generally considered as diploids but, from evolutionary point of view, being actually diploidised polyploids (after polyploidisation event).

The 5S rDNA clusters in fishes seem to be most frequently located at interstitial chromosome sites as they were found in most fish species in different orders (Martins and Wasko 2004). A non-terminal location of this rDNAs could reflect an ancestral condition of the chromosomal organisation (Martins and Wasko 2004, Nakajima et al. 2012). The 5S rDNA loci observed in the karyotype of *Carassius carassius* (Fig. 1e) near the centromere region and in a subcentromeric position as well as similarly located such loci in the karyotypes of *Carassius gibelio* and *Carassius auratus* (Zhu et al. 2006) may reflect chromosomal rearrangements. The activities of repetitive sequences as well as transposable elements are often correlated with genomic sequence elimination and chromosome rearrangements (Zhu et al. 2006). However, additional discrete signals after FISH with 5S rDNA probe may appear as a result of hybridisation to the chromosome regions consisting of repetitive sequences similar to the 5S rDNA fragments (Ferreira et al. 2007).

In most of the described fish species including cyprinids, the two rDNA families are located at different chromosomes (Fujiwara et al. 1998, Singh et al. 2009, Nakajima et al. 2012; Kumar 2013). However, in others, including some cyprinids, the minor rDNA loci are co-localised with the major rDNA loci in the same chromosome (Inafuku et al. 2000, Gromicho and Collares-Pereira 2007, Pereira et al. 2012). In *Carassius carassius* as well as in *Carassius gibelio*, the minor and major rDNA clusters are located in different chromosomes (Zhu et al. 2006). This 5S rDNA pattern, with none of the numerous signals overlapping with the 28S, represents a characteristic cytogenetic feature of *Carassius* species.

## Conclusion

We updated the information on the karyotype, showed for the first time sequentially CMA_3_/AgNO_3_ banding pattern and also provided new molecular cytogenetic data on the crucian carp *Carassius carassius* using double-colour FISH with 5S and 28S rDNA probes. The obtained results improve our knowledge about the chromosome structure and physical location of major and minor ribosomal sequences in this fish species. Moreover, the results herein gave an important insight into the molecular cytotaxonomy of the crucian carp a polyploid and declining species and may be useful in its systematics.
